# Community led active schools programme (CLASP) exploring the implementation of health interventions in primary schools: headteachers’ perspectives

**DOI:** 10.1186/s12889-015-1557-0

**Published:** 2015-03-13

**Authors:** Danielle Christian, Charlotte Todd, Helen Davies, Jaynie Rance, Gareth Stratton, Frances Rapport, Sinead Brophy

**Affiliations:** College of Health and Human Science, Swansea University, Wales, SA2 8PP UK; College of Medicine, Swansea University, Swansea, SA2 8PP UK; A-STEM College of Engineering, Swansea University, Wales, SA2 8PP UK

**Keywords:** Qualitative, Interviews, Headteachers, Health interventions, Physical activity, Primary school, Children

## Abstract

**Background:**

Schools are repeatedly utilised as a key setting for health interventions. However, the translation of effective research findings to the school setting can be problematic. In order to improve effective translation of future interventions, it is imperative key challenges and facilitators of implementing health interventions be understood from a school’s perspective.

**Methods:**

Nineteen semi-structured interviews were conducted in primary schools (headteachers n = 16, deputy headteacher n = 1, healthy school co-ordinator n = 2). Interviews were transcribed verbatim and analysed using thematic analysis.

**Results:**

The main challenges for schools in implementing health interventions were; government-led academic priorities, initiative overload, low autonomy for schools, lack of staff support, lack of facilities and resources, litigation risk and parental engagement. Recommendations to increase the application of interventions into the school setting included; better planning and organisation, greater collaboration with schools and external partners and elements addressing sustainability. Child-centred and cross-curricular approaches, inclusive whole school approaches and assurances to be supportive of the school ethos were also favoured for consideration.

**Conclusions:**

This work explores schools’ perspectives regarding the implementation of health interventions and utilises these thoughts to create guidelines for developing future school-based interventions. Recommendations include the need to account for variability between school environments, staff and pupils. Interventions with an element of adaptability were preferred over the delivery of blanket fixed interventions. Involving schools in the developmental stage would add useful insights to ensure the interventions can be tailored to best suit each individual schools’ needs and improve implementation.

## Background

During childhood, behaviour patterns are established which have important implications on the short and long term health and well-being of children [1]. Children spend a significant amount of time in school creating a key opportunity to reach a wide range of children across the population, regardless of social background [2]. As a result, schools are often seen as key settings for health promotion for a number of public health interventions [3].

Multi-component interventions within the school setting seem to be most effective, particularly on outcomes such as physical activity, fitness and adiposity [4-6]. However, others have found mixed results regarding the effectiveness of school based interventions [7,8]. These studies have provided limited information on process and implementation factors making it difficult to determine why some interventions have been successful and others not. This information is invaluable for informing future interventions.

Helping schools to extend their role in health promotion could be a key driver in improving public health [9]. Historically, health promotion initiatives have been developed using a ‘top-down’ approach, which have yielded mixed results. However, the reverse approach may be more sustainable as developing programmes with key stakeholders at ground level will make commitment and support of the intervention more likely, resulting in more effective implementation [10]. Thus, in order for school-based health initiatives to be effective and sustainable, it is crucial to engage with school stakeholders who have key insights into the barriers and facilitators to implementation [11]. Headteachers are considered the cornerstone of primary schools. Therefore, gaining their views can help researchers understand how to best tackle both current and future health problems and inequalities from a school’s perspective.

Previous qualitative research with school staff and pupils highlighted a number of influential factors which affect implementation of school based health interventions including funding, environmental factors, competing priorities and confusion over responsibility between schools and parents [12]. In light of these barriers, Stolp [13] identified stakeholder buy-in and the provision of adequate resources as two key elements that must be addressed when developing healthy school communities, whereas Jones [14] focused on six key areas for consideration for implementation: design and analysis; school-community engagement; planning and recruitment; evaluation; implementation; and feedback and sustainability. However the majority of these studies were from the United States, Australia or Canada and it is likely that there may be differences in barriers faced in the United Kingdom (UK) due to differences in schooling systems. Whilst there has been research exploring the barriers to physical activity in adolescents in the UK [15], there has been little exploring the views of headteachers regarding school-based health initiatives in primary schools and how to address these when developing future interventions.

### Aim

To explore headteachers’ views and experiences regarding school-based health interventions, with particular reference to identifying factors that could facilitate implementation of future complex school-based interventions.

## Methods

### Ethics statement

Ethics approval was granted by Swansea University College of Human and Health Sciences Research Ethics Committee (CHHS REC) (October 2012). All participants provided informed written consent prior to participating. Personal data generated from this study was anonymised using unique identification numbers and all paper data was securely stored in locked cupboards and electronic data in password protected files on a secure university server. Paper data was kept separate to identifiers at all times.

### Sampling population and recruitment

Purposive sampling was used to recruit headteachers for this study. All 84 primary schools in Swansea were contacted detailing the study aims and protocol. Following expression of interest, a date for an interview was arranged. If the school did not wish to participate, this was noted and there was no further communication regarding the study. In the event of non-respondents, a final phone call was made and another non-response at this stage was accepted as a lack of interest in participation in the project.

### Data collection

This explorative study used qualitative semi-structured interviews [16] with a grounded theory approach [17]. Interviews were chosen as they allow a rich and deeper understanding of participants’ views which is particularly insightful in an explorative study such as this [18]. The views of all those who wished to participate were incorporated. A semi-structured topic guide was designed to aid discussion into the challenges from past and current health interventions and recommendations for future school-based designs. Example questions include: What impacts has health research or interventions had on the school? Is there anything that could be done to lessen or amplify these impacts? What attracts you to current health initiatives? Semi-structured questions were used to allow topics to form more naturally during the interview process and the topic guide was initially validated through two pilot interviews.

Participants were interviewed individually bar the exemption of two interviews where headteachers requested a school health co-ordinator to also be present. Each interview was conducted by two researchers (DC and CT or DC and HD). One researcher (DC) facilitated the interview process whilst the second (CT or HD) provided technical support (digitally recorded) and noted the interaction between interviewer and interviewee. The second researcher then verbally summarised the key points back to the interviewee at the end of the interview to either question, clarify or give further insight. This use of respondent validation ensured that the researchers had a good understanding of the participant’s views reducing the chance for misinterpretation of results or potential bias [19]. These observations were further discussed post-interview to allow for progressive adaptations to the topic guide, in accordance with the iterative grounded theory approach [20]. Interviews were carried out between January and March 2013 and took place in the school setting.

### Data management and data analysis

Each interview encompassed interaction and responses between participants and moderator which were digitally recorded and transcribed verbatim in Microsoft Word. Each verbatim transcription then served as a primary text document. Using qualitative thematic analyses [21] each primary document was independently read several times by (DC and CT or DC and HD) to gain theoretical sensitivity which emphasises the participants’ frame of mind [22]. The primary researcher (DC) used open coding, whereby a word or phrase was assigned to each quote, conversation or paragraph in an attempt to encapsulate the participants’ meaning. A second researcher (CT or HD) independently coded the transcripts in the same manner and then checked the codes for accuracy and consistency with the primary researcher (DC). Agreement between coders was high but any discrepant codes were discussed and agreed upon with a third researcher (CT or HD, alternate to second researcher). Related and reoccurring codes were grouped together to form main themes, for example codes such as ‘school’s agenda’, ‘school philosophy’ and ‘school values’ were all brought together under the theme suggesting interventions need to be ‘supportive of school ethos’. Specific quotations from the primary documents were then collated to illustrate the salient themes. Any outstanding methodological or analytical discrepancies were further verified at a tertiary level via an external qualitative lead to ensure voracity throughout.

## Results

19/84 (23%) primary schools agreed to take part. 13 (15%) declined to participate; 9 (11%) due to busy workloads and an overload of initiatives, 2 (2%) due to impending Estyn inspections and 2 (2%) declined to comment. 42 (50%) schools did not respond and 8 were busy at the time of calling. One school sent their opinions via email. The 19 participants who consented to take part included 16 headteachers, 1 deputy headteacher and 2 healthy school co-ordinators. 7 were male and 12 were female. The participating schools ranged in deprivation scores from 3% to 53% free school meal eligibility (Mean = 22%) which was identical to non-participating schools (22%).

### Challenges to implementation of health interventions in schools

The main challenges restricting implementation of interventions reported by headteachers were government-led academic priorities, initiative overload, low autonomy for schools, lack of staff support, lack of facilities and resources, litigation risk and parental engagement.

### Government-led priorities and funding

The main challenge highlighted by many participants was that the current Governments’ priorities for schools are to improve literacy and numeracy for all; hence educational achievement has been dictated as the school’s main priority (see Table 1). Schools feel they are predominantly assessed on educational achievement and not on health of the child so time spent on health reduced valuable curriculum time, leaving participants feeling overburdened. Until schools are measured on, funded and recognised as providers of health and social development, health will always be seen as a secondary priority for teachers, behind academic achievement (see Table 1).

**Table 1:** **Challenges of implementing health interventions in primary schools**

**Government-led priorities and funding**	“We know that physical activity is hugely important but when we’re actually getting measured by the Welsh Assembly Government on our performance in literacy and numeracy, you can tend to push physical activities out to one side…”(Participant E - Headteacher)
	“It’s English and maths you know, we’re being hammered, English and maths, English and maths, that’s all that counts, and the Olympics come along, well sports important, or obesity comes up, sports important, it’s not really because there’s no extra funding in it you know…” (Participant N - Headteacher)
**Initiative overload**	“We have the Welsh Assembly Government giving us initiatives, we have regional giving us, we have then our Local Authority giving us initiatives. We have then other things like we’re doing rights respect in school, we’re doing restorative practice, we do valley’s education, we do Healthy Schools, we do sustainability, we do a European schools, we do all these things, so yes, we do feel burdened.” (Participant A – Headteacher)
	“They want us to manage their agenda for them, they don’t really… they’re not terribly bothered about ours…I mean, [X] will ring up at the end of a term and say, ‘Oh, how many children have taken part in after school clubs this term? We need the figures. And you just feel… I mean, that’s it, though, isn’t it. You need the figures. It’s a data crunching exercise. It’s got very little to do with you actually coming out and seeing if there’s any quality in that activity.” (Participant J - Headteacher)
**Autonomy v statutory approaches**	“I mean obviously there are some interventions which are statute and we’ve got to and there’s no choice I’m afraid, but I think it’s those, (coughs) excuse me, that sometimes, well it’s because of those that the more exciting, more creative activities don’t happen if you like, because of the legislative, the ones that we have to do that are statutory requirements.” (Participant H - Headteacher)
	“…we don’t have that power as a school. We’re a recipient, if you like, rather than a leader.” (Participant J - Headteacher)
	“I think sometimes there, that’s taken away from us in terms of that expertise because I know what works in my school isn’t necessarily going to be relevant in the school next door so it’s that lack of trust really that ‘just leave us alone to do it’ and yes, of course we’re accountable and I wouldn’t want to take any of that accountability away, but just let us get on with what we’re doing because it, we’re making it work for our children and you know, we’re not the experts but we do know what we’re doing.” (Participant H - Headteacher)
**Health and safety litigation**	“But I did have a parent come in ‘cos we had some stepping stones made out of pieces of wood and they’d have little bit of fungus growing on the side and parents saying, ‘They shouldn’t be out there, that’s a health hazard that is,’ and I’d say, ‘Well no, they’re okay’, and again you’d have to tough it out sometimes and take it like the rest of it, because if the pieces of wood get wet and the children are jumping from one to the other they get slippery, they fall off and they learn then, it’s no good trying to play stepping stones when the wood is all wet, you know, we’ll do that when it’s dried out.” (Pilot 1 – Retired Headteacher)
	“Well they can’t, they couldn’t just go into the gym break time ‘cos it’s break time, you know, it has to be supervised and you know, we don’t tend to have an after school for our infants because obviously you’re staffing ratio gets higher, you know…” (Participant K - Headteacher)
	“We do things, we’ve taken the children to London and, you know, as long as you’re confident as a staff that you’ve risk assessed, you know exactly what you’re doing, the staff are all briefed, the ratios okay, you know, and we’ve just tried to carry on, because at the end of the day you want to enrich the children’s education, don’t you, you don’t want to sort of narrow it down, but health and safety is a nightmare, yeah.” (Participant D - Headteacher)
**Staff and headteacher influences**	“Our football, Mr [X] you just met in there, he takes the football club and it’s basically in his own time and he is, you know, he’s fantastic, he’s a real sort of football enthusiast and that rubs off on the children because they’re very successful in football.” (Participant Q – Deputy Headteacher)
	“…the previous headteacher was a heavy smoker (laughs). So he wouldn’t even let the Healthy Schools Coordinator in through the door. So (laughs) this was like the jazz club in here, it was just smoke filled…”(Participant D - Headteacher)
**Physical environment and facilities**	“It’s almost as if we’ve kind of got it backwards in this country because if you go to any university campus you will generally have very good sports facilities, especially if they’re offering a sports science kind of degree, you’ll have out of this world facilities. Go backwards towards the comps and you’ll kind of get reasonably good facilities, a lot of comps have got gyms, they’ve got big, you know, indoor halls. But then as you go down to primary schools you’ve got, usually the school hall, that’s usually for cooking as well and you know, for assembly and for everything else…” (Participant I – Healhy School Co-ordinator)
	“Yeah, I think you just need to be quite… you need to have a plan, basically. And what we’ve found is discipline goes, behaviour goes at play time if there isn’t anything structured. So our children, they get to play football at the front every break time, sometimes if it does go a bit too far we do have to say, ‘Well, look, you’ve had your yellow card now and if it carries on there’ll be no football tomorrow,’ and we do have to stop it sometimes.” (Participant L - Headteacher)
	“The only thing that really, I think the barriers to that quite often are your consumable equipments so, you know, balls will go over the gardens and the skipping ropes get sort of manky and disgusting so it’s having a regular supply really of equipment because school budgets are very tight but that’s another issue for us, you know, if you’re talking about constraints.”(Participant E - Headteacher)
**Parental engagement**	“Parents are more of a problem than the children perhaps… So it's educating parents and getting through to them because they seem to be the barrier. You know you educate the children and they seem to understand and they can sort out healthy and non-healthy foods but it doesn't, the message doesn't seem to get home so it's parents and actually getting the parents in to school. Some of them are very you know not really interested, some are some aren't, you know it's the same isn't it?” (Participant M - Headteacher)
	“If you want to effect the parents then you need to get access to the parents and you need to get access to them in an informal way, and I’m sure then they come onboard…you’ve got to go say through children to places like children’s centre, which are non-threatening, yeah, which they feel comfortable going there, it’s their choice to go there, do you see what I mean, they’re there to offer support and help and I think that is a good way of reaching them.” (Participant G - Headteacher)

Although funding is available, it was believed this was limited and restrictive meaning participants often opted for free activities rather than those needing investment by the school. In schools in more socially deprived areas where extra funding was available, this was often not sustained. These schools felt once they had invested an interest and managed to successfully implement a programme, the funding was often pulled by the government for other priorities.

### Initiative overload

Participants commented that they were often inundated with new ideas and initiatives which were short term or unsustainable. These initiatives stem from Government, local authority, public health, universities and charities who often work separately or are unaware of each other’s agendas. Some headteachers remarked on this lack of collaboration with health schemes appearing ad hoc, haphazard, and disengaged from children and schools. Furthermore, few suggested that many initiatives feel like number crunching exercises in order to appear to do something for purpose of award rather than assessing quality and sustained investment in children’s health (see Table 1).

### Autonomy v statutory approaches

The enforcement of statutory programs by the Government often left teachers with limited say in what works best for their school or ways of improving implementation locally. There was an interpretation by participants that schools sometimes had no say or no choice and just had to do what they were told, even if they knew it was unlikely to work in their school. It was felt that with no ownership or input in the programme, generic ideas often did not work at a local level. Furthermore, mandatory government-led projects were perceived by some to take up time which could be given to more creative local projects allowing schools greater autonomy over implementation.

### Staff and headteacher influences

The headteachers reported how integral they themselves were in the implementation of interventions within the school. Headteachers who were positive about being healthy, and engaged in healthy behaviours themselves, were thought to be paramount in influencing and engaging pupils as well as maintaining health-based initiatives. Equally, staff who engaged in unhealthy behaviours were thought to have a negative influence and were less likely to prioritise health.

Many interventions actually required very engaged motivated staff who dedicated their own time to maintain adherence and promote success. In fact, the commitment of certain members of staff was repeatedly applauded. However, one headteacher suggested that due to the increased dependence of technology within education, teachers with Information and Communications Technology (ICT) skills are likely to be valued more than those enthused by physical activity and health. This move of priorities becoming ICT focussed rather than health may mean health interventions slide further down the priority list for schools.

### Health and safety litigation

A real barrier to allowing imaginative free play recurrently mentioned was the potential for litigation issues and the need to follow clear health and safety and risk assessment guidelines. Accountability for injuries or accidents often meant teachers did not want to run activities where injuries were more likely to occur. Participants expressed a requirement to adhere to staffing ratios, thus monitoring children at all times to ensure correct safeguarding for the pupils. These restrictions meant children had little opportunity to experience unsupervised free play during the school day, especially for infants who require even higher staffing ratios.

However, there was the feeling among some headteachers that children do need to learn to assess risks and equally make mistakes to learn through play effectively. Therefore health and safety guidelines, whilst not perceived as completely prohibitive, did mean that any activities need extensive pre-planning, risk assessments, parental consent, correct staff ratios and staff briefings to minimise risks. Litigation was also a barrier for active transport to and from school due to risks on local roads and unsafe communities.

### Physical environment, facilities, resources and weather

The physical environment and facilities varied dramatically from school to school, from small inter-city schools with just one small school yard to rural schools featuring extensive school grounds. Some participants felt this restricted opportunities to bring in outside specialists to run physical activities. One participant even went as far as to say they felt that the actual primary school environment was no longer conducive to demands and greater investment was warranted to match the latest state of the art facilities present in secondary schools and universities.

Headteachers noted that some children lacked the ability to play in a restricted space which when combined with a lack of skill and knowledge was believed to lead to fighting and other behavioural problems. Some headteachers even enforced school policies on physical activity (such as no ball games) due to the restricted space and health and safety risks.

Bad weather appeared to exacerbate these issues as for many this prevented pupils going outside for free play; however, the magnitude of this restriction seemed to come down to teacher’s discretion regarding risk. Some schools encouraged outdoor play in the elements, whilst others felt the health and safety risks were too great and children needed to stay inside. This was perceived as a greater barrier for girls who were unwilling to get wet and cold outside.

It was recognised that children needed resources and equipment to play with, but these consumables quickly needed to be replaced when broken or lost. Indeed, some schools commented on children’s lack of care for consumable equipment meaning light equipment was often lost over walls or damaged within a short time frame. The replacement was a luxury not all school budgets could afford repeatedly. One school pointed out that they did have equipment but there had to be someone interested in repeatedly buying, maintaining and removing old equipment. Furthermore, storage was a concern for some schools with regards to consumable equipment.

### Parental engagement

Many participants spoke of the need for parents to be involved in health schemes as parents are largely responsible for the reinforcement of positive health behaviours in the home. One participant proposed the need to educate the parents with regards to key health issues and that a greater partnership between schools and families was needed to influence change. However parental input can differ wildly ranging from heavily supportive to completely disengaged with health and it is difficult to try and engage equally across the spectrum.

### Guidelines to creating successful health interventions within schools

**Figure 1:**
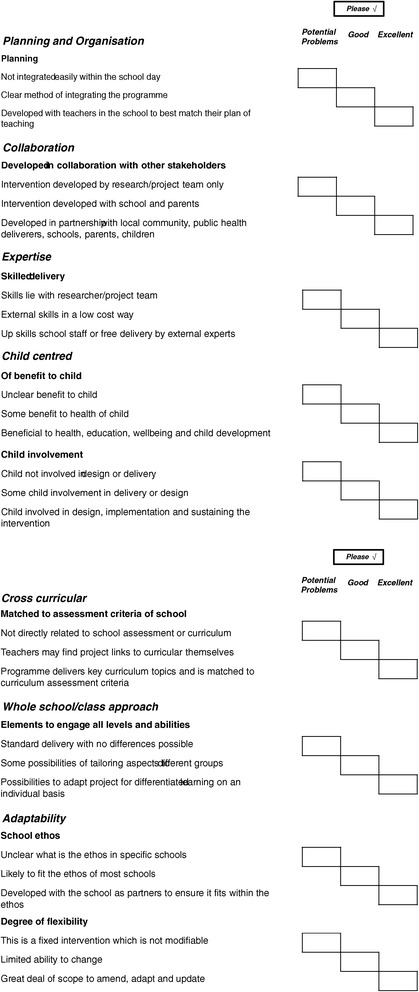
**Checklist for approaching schools with new interventions.**

Not all the challenges expressed above can be addressed straightaway, such as the governmental policy focus or the limitations of school settings. However Headteachers reported a number of recommendations that can be utilised in the immediate future. These guidelines were taken forward to formulate a guidance checklist to be utilised by those developing future school-based health interventions (Figure 1). Inherent to the recommendations below and highlighted throughout all interviews was an overarching theme of sustainability. Indeed, many commented that health initiatives need to have built in measures to ensure they are sustainable after the initial intervention period.

### Planning and organisation

Participants requested a need for interventions to be well-planned, well-packaged and organised and it was suggested close contact with researchers or personnel delivering the project would help gain the most out of the project (Table 2).

**Table 2:** **Guidelines to creating successful health interventions within primary schools**

**Planning and organisation**	“Where it’s packaged and well thought out and well supported, you know, that’s when you get the benefit…" (Pilot 2 – Retired Headteacher)
	“…also that we know in advance that you’re coming in because if the class teachers plan to do something and then only two or three children are coming out, then that is destructive because one they’ve missed whatever it is the class is doing, and secondly after you are finished with them and they come back in the class teacher then has got to get them into the lesson and catch up on what they’ve missed. So it’s about organising the time that you do come in.” (Participant F - Headteacher)
	“…what we find is that the government tend to fund something and when it’s working really well, they take the funding away…” (Participant E - Headteacher)
**Collaboration**	“I think it’s collaborating with sports clubs. I know that rugby has got this phrase, ‘The club is the hub’, and really these days the clubs are the people who are driving physical activity, and we just give them the tasters really. I think where we do our best work we probably are linked in with clubs.” (Participant P - Headteacher)
	“I feel strongly that you need to get everybody on board so you need to have the child themselves understanding, right, this is what’s going on and this is why it’s going on, you need to have the parents on board because unless they’re on board and they understand exactly why we’re doing what we’re doing then they could potentially undo some of the hard work that’s been put in…the class teacher needs to be involved so that they can reinforce it with the children, and then whoever’s delivering it so whether that would be health professional or yourselves, somebody from the university, you know, so it’s lots of people involved in it.” (Participant C – School Health Co-ordinator)
**Expertise**	“I think what we do tend to lack is expertise, because everybody kind of thinks ‘oh yeah, you can go and do a gym lesson, that’s not a problem at all’ but in actual fact, our staff are not experts…” (Participant E - Headteacher)
	“Well ideally it would need to be sustainable so if somebody came in and up-skilled our staff and worked alongside them for a period of time and then left so it could be maintained because we understand, you know, you can’t be every day for whatever so there would be an element of sort of training with them, modelling it to them, leaving and then having sort of visits every now and again, coming back, making sure that what was set up is still in place.” (Participant Q - Deputy)
**Child-centred**	“…so it’s finding out what the children want so putting questionnaires out prior to starting it, to find out exactly what they would come to in terms of physical exercise so maybe giving them a choice of, I don’t know, ten different things and saying, which one would you turn up to so that they’ve got some kind of input in it and they’re far more likely to come if they think they’ve had a hand to play in it as well…” (Participant C – School Health co-ordinator)
	“…we’ve found things that the children are involved in are the most successful…it’s just part and parcel of what we do, it’s not even given a second thought and that’s the sort of thing you want isn’t it that when you move and they take it over, that it will run and be sustainable…” (Participant G-Healthy School Co-ordinator)
**Cross-curricular**	“Coz when it snowed we were open, went out in the snow and we did like welsh describing words, you know they actually held the snow, that's the best way to do it rather than sitting here and do it so you know go out and give them firsthand experience of it. They learn better like that.” (Participant M - Headteacher)
	“…it needs to be agreed focus but also it needs to match our assessment criteria of the children because very often we have for instance, organisations come in, they’ll do like a block of activities and then they’ve gone but then we have no sort of understanding of what skills they’ve hit, how that matches our assessment of the children so then that becomes a reluctance because that’s taken up perhaps four or five weeks of our sort of PE time or games time with the children and we’ve got to go back and reassess and so on so there needs to be that link as well…" (Participant Q – Deputy Headteacher)
**Whole school or whole class approach**	“So yeah, where it has a significant impact on children’s learning, I like, as I say, whole school and whole phase, those that are inclusive, we have two specialist teaching facilities here for children with moderate learning difficulties and we have a number of children with mobility issues or difficulties and so everything we do we’re always considering how can we include everybody in this, and that’s one of the wow factors, if you like, when you sort of stand back and reflect or observe something is seeing the children working in harmony with each other and being very accepting and supportive, excuse me, so that’s something that’s really important to us here as a school.” (Participant H - Headteacher)
	“…we will offer the children the chances to take part in a competitive way, but only if they want to. It doesn’t count. The points count for the thing everybody takes part in. Those events are just there because some children are good at it, but nobody’s gonna have to run and come last openly. I mean, you’d never do it in any other curriculum, so why would you dare humiliate kids, you know, it’s horrible isn’t it. It’s what I grew up in. I was lucky, I was quite good at athletics, but for kids who… There couldn’t have been anything worse.” (Participant J - Headteacher)
**Adaptability**	“Can I say perhaps that academic researchers sometimes have no idea what goes on a school, at the you know, at the 200 screaming kids level.” (Pilot 2 – Retired Headteacher)
	“I think there’s a lot going on, yeah, I don’t know that there’s too many, I think there just seems to be a huge period of change if you like but the change is very quick, no sooner have you started to embed something then another thing comes along and it gets a little kind of like right, whoa we’re gonna stop, we’re gonna focus on this, this is what we’re going to do and we’re going to embed it, ‘cos it’s right for our school, and not every intervention is right for every school, you know, there are some that are more needy in some areas than others and I think it’s sort of acknowledging what is right for your school and thinking yeah, this is the path we’re going to go down.” (Participant H - Headteacher)
	“it’s always about reinventing things and teachers are pretty good at thinking outside the box and they can be fairly creative. So I think it’s about putting new twists on things, really, to be honest, just to keep everybody into… and you can tell when things start to flag, can’t you, you know.” (Participant E - Headteacher)

Some participants believed parents could be anxious regarding interventions or research projects, though this could be lessened if they were fully informed from the outset. As well as a high level of communication, reliability from those delivering the project was imperative to prevent school disruption and pupil disappointment, as this could result in the project being dropped indefinitely. Realistic outcomes needed to be factored into planning and it was felt reviewing progress during the intervention would maintain interest. However for evaluation to work effectively, headteachers expressed a need for a member of staff at the school to take lead to co-ordinate processes and organisation.

The cost or financial component is also an important aspect that needs to be addressed during the planning stages. Initially free interventions that then require a school to fund ongoing costs or transport children to facilities will not be sustained if they are not cost effective, feasible or offer the school value for money.

### Collaboration

Many participants saw the advantages of collaborating with partners within different health disciplines or in some cases strengthening those that were already present. Health visitors, sports development officers and local sports clubs were amongst those named for opportunities to strengthen partnerships. Indeed, this would help overcome the issue of overload experienced as a challenge in implementing school-based health interventions. One healthy schools co-ordinator even suggested all disciplines collaborate together to allow schools to assess all aspects of the child.

Parental and community involvement was also seen as important to ensure collaborative working towards common goals or agendas whilst at both school and home. Although engaging parents can be difficult, it was felt if there was some perceived gain from parents they may be more receptive. Headteachers suggested that informal methods were more effective, such as utilising school performances and the school gate pick up and drop off times positively to informally engage. These approaches may also need to stretch to child minders, crèches and other carers who have a significant influence on the child’s daily life.

### Expertise

Participants felt that there was a lack of expertise regarding health and physical activity amongst staff unless they had undergone specific training or had experience in this area. It appeared to be an issue that wasn’t addressed during teacher training therefore heavily relied on the experience of the teachers themselves. As a result, expertise was a favourable attraction when assessing potential interventions, which would often be found through externally-led initiatives. Whilst this was perceived as advantageous in raising engagement and offering inspiration to pupils it was noted that externally-led interventions are not always sustainable. Hence, adding a staff-development aspect to the intervention for existing school staff members was seen as a way of addressing this. Up-skilling staff would provide them with the knowledge and education needed to continue working in these areas post-intervention enabling the intervention to be maintained. However, staff development also presented a challenge with some teachers suggesting that this requires supply cover to be obtained, another added expense to schools tight budgets.

### Child-centred approach

The majority of participants made reference to a child-centred approach suggesting it was imperative that the intervention was beneficial to the child in some way. Whilst they expressed the constant pressure due to other demands on the school, they would be willing to make exceptions for clear and obvious benefits for the children; especially for their happiness, motivation and learning. It was felt that interventions that allowed pupils to have an active role in implementing and maintaining interventions were likely to be more sustainable. Most importantly the intervention needed to be engaging and child focused so children want to take part. Provision of opportunities for practical, hands-on experience was also considered a highly positive point of interventions by some participants, as well as making an event of initiatives and linking interventions in with current trends.

### Cross-curricular approach

Participants suggested interventions should be fully-integrated and shouldn’t be viewed as discrete entities but should link between different parts of the curriculum. It should be clearly illustrated how health initiatives are cross-curricular and how they match the assessment criteria for the schools. This is especially important due to the tight time constraints of the curriculum and the amount needed to be covered during the academic year.

### Inclusive whole school approach

Whilst some teachers saw the need to sometimes use targeted approaches, it was generally agreed that activities that only target a specific group of pupils were seen to be disruptive to learning. Most participants preferred activities that tended to include a whole class or whole school as this made it easier to manage effectively within a school environment. With the range of differing abilities at schools, an element of inclusiveness encompassing all children was seen as an attractive element of an intervention, especially if it complemented the school’s values. Interventions regarding physical activity need to contain elements to capture the attention of disengaged pupils as activity-based interventions tend to appeal to already active pupils. Competitiveness can be a barrier to physical activity and potentially detrimental to the welfare of some pupils therefore offering a range of competitive and non-competitive aspects was suggested to enhance an inclusive approach.

### Adaptability

Participants reported sustaining interventions as challenging as it was hard to maintain the same levels of enthusiasm over time. Refreshing or reinventing projects or ideas was seen as an effective way of combating this which needs to be factored in when designing interventions. A flexible approach was also deemed useful due to the dynamic nature of primary schools and would appeal to headteachers as they could adapt the intervention to fit around their prior plans.

Interventions need to complement the schools’ values and day to day operation and be planned well in advance of the date of implementation. Those which do not attempt to understand the day to day operation of the school were perceived as more likely to fail. Often the school curriculum is planned up to a year in advance and new initiatives cannot always be implemented at short notice. Interventions should be designed in partnership with schools allowing greater input ensuring it fits the schools’ ethos and values. This means clear methods of communication between researchers, project workers, teachers, parents and all involved are needed.

### School’s agenda for future health interventions

Topics that many schools mentioned as important areas they would like addressing in the future, either individually or as part of a larger multi-component intervention, were; mental health, play (especially for girls and at playtimes), active transport and improving behaviour. However, interventions to target these aspects of health would need to take on board the barriers to working in schools and would need to meet recommendations such as those given in Figure 1 (Guidelines to improving school-based interventions).

## Discussion

This study found that whilst headteachers in primary schools appreciated the importance of child health, implementation of health interventions were hindered by priorities of educational achievement, lack of funding, initiative overload, risk of litigation, lack of facilities and low parental engagement.

The issue of government priorities and curriculum pressure has been identified previously as a large barrier when implementing school based health interventions [23]. Pressure to prepare students academically limits the amount of time schools are able to set aside to deliver health related activities. Thus, it has been argued that schools need to be recognised and funded on the work they do for children’s health and well-being as well as for educational achievement. For health interventions to succeed, they need to be prioritised in educational mandate at a local and national level and funding needs to be available to support the interventions [2,12,24]. In addition, initiatives need to be developed with long-term investments in mind as short-term projects often change repeatedly and lead to initiative overload.

The physical environment of the school is an area which has attracted considerable research attention in attempting to improve child health [25-28]. There is a growing concern from parents and teachers that challenges in school environments, as well as facilities, weather and resources can restrict opportunities for unstructured, free play opportunities and consequently reduce young people’s ability to play [29]. Indeed, provision of equipment and improvement of physical environment alone may not impact on physical activity levels if children do not know how to play in the first instance.

Concerns related to injury risk and litigation were commonly discussed as barriers to implementing physical activity interventions. In order for physical activity interventions to be fully embraced, parents and teachers need to be willing to accept risk of injury. A recent intervention involving an educational component teaching parents and teachers about risk, has shown promise and warrants further investigation [30].

Parents can play a key role in influencing their children’s physical activity and health behaviours [31]. However, the majority of participants described parental engagement with the school regarding health as poor. Participants talked about parents being part of the problem but felt there was also a role for parents in improving children’s health. Involving parents in school-based health interventions was expressed by the majority of participants as challenging. This difficulty has been expressed by teachers in a recent study [24] and no consensus has emerged in previous research or indeed in this study, regarding the best way to involve parents [32,33].

Nevertheless, a large amount of participants in this study highlighted the need for shared responsibility as headteachers and school staff can also be influential. Similar beliefs have been expressed by staff in recent research where teachers described themselves as important role models in influencing children’s behaviour [23]. Furthermore, social support from teachers was shown to be a significant mediator of behavioural change in the Fit 4 Fun intervention looking to improve physical activity levels in primary school children [34]. Therefore it is important to ensure a joined-up approach to improving the health of children reinforcing the importance for stakeholder buy-in and effective partnership working as expressed by Stolp [13].

Alongside highlighting the challenges faced, headteachers provided a number of recommendations for implementing school based health interventions such as collaborative working and whole school approaches, which have been highlighted in previous research [9,12,24]. Indeed, the need for effective partnership working between those delivering public health interventions will be crucial, especially in current times when budgets and resources are limited. Collaboration in this manner will ensure projects are delivered with the desired expertise and school staff can be supported and developed effectively for sustainability of projects: a major theme featured prominently throughout the interviews. However flexibility was expressed as key to this process, not to mention the project implementation itself, mirroring the thoughts of Jones [14] who went as far as to say it is ‘required for contextual relevance and responsiveness in changing school circumstances’.

Research in the school setting should consider cross-curricular approaches and educational outcomes, as demonstrating a positive effect on educational outcomes may increase the likelihood of teachers adopting interventions. Indeed, if schools can identify benefits to an intervention that fit in with their key curricular outcomes, the separation that exists between health and education will be reduced [35]. A consideration of school ethos is also important to consider when planning interventions [36].

These recommendations were taken forward to formulate guidelines for those looking to implement primary school-based interventions. Checklists have been used in the past in order to inform those looking to ensure projects are youth-friendly [37]. However to our knowledge this is the first set of guidelines designed by headteachers to inform those specifically designing primary-school based health interventions.

### Strengths and limitations

This qualitative study allows a rich exploration of the process and implementation factors influencing why some health interventions may be successful and sustainable within primary schools and others may not. It is unique in adding to the minimal UK based literature on headteachers’ views of school-based health interventions. Indeed, the inclusion of these key stakeholders in this study allows for pragmatic insights which are invaluable for those designing primary school-based interventions. The use of respondent and expert validation increases the rigour of these findings. However, as is the nature of qualitative work it should be noted that these findings cannot be generalised to all primary schools. Also, headteachers volunteered to take part so there may have been a selection bias introduced with recruited participants having a greater interest in health and wellbeing. Additionally limited data was gathered regarding participant’s age, culture, and years of teaching which we appreciate would have given useful insights. However, with this small sample size there would have been too little information to ascertain any differences between these factors.

## Conclusion

Whilst schools may be instrumental in improving the health of children, there are a great deal of challenges compromising the effective implementation of health interventions within school settings. Researchers and health practitioners should develop interventions which take on board the schools perspective, such as those given in Figure 1. Interventions should recognise these possible challenges and recommendations to further enhance success. Involving headteachers and key stakeholders during the intervention development stage may be crucial to implementation success and intervention longevity.
